# A distinct transcriptional signature of antidepressant response in hippocampal dentate gyrus granule cells

**DOI:** 10.1038/s41398-020-01136-2

**Published:** 2021-01-05

**Authors:** David P. Herzog, Diego Pascual Cuadrado, Giulia Treccani, Tanja Jene, Verena Opitz, Annika Hasch, Beat Lutz, Klaus Lieb, Inge Sillaber, Michael A. van der Kooij, Vijay K. Tiwari, Marianne B. Müller

**Affiliations:** 1 Department of Psychiatry and Psychotherapy, Johannes Gutenberg University Medical Center Mainz, Mainz, Germany; 2 Focus Program Translational Neurosciences, Johannes Gutenberg University Medical Center Mainz, Mainz, Germany; 3 Institute of Physiological Chemistry, Johannes Gutenberg University Medical Center Mainz, Mainz, Germany; 4 Institute of Microscopic Anatomy and Neurobiology, Johannes Gutenberg University Medical Center Mainz, Mainz, Germany; 5 Genevention GmbH, Göttingen, Germany; 6 Institute of Molecular Biology, Johannes Gutenberg University Mainz, Mainz, Germany; 7 Wellcome-Wolfson Institute for Experimental Medicine, School of Medicine, Dentistry & Biomedical Science, Queens University Belfast, Belfast, UK

**Keywords:** Depression, Pharmacology, Molecular neuroscience

## Abstract

Major depressive disorder is the most prevalent mental illness worldwide, still its pharmacological treatment is limited by various challenges, such as the large heterogeneity in treatment response and the lack of insight into the neurobiological pathways underlying this phenomenon. To decode the molecular mechanisms shaping antidepressant response and to distinguish those from general paroxetine effects, we used a previously established approach targeting extremes (i.e., good vs poor responder mice). We focused on the dentate gyrus (DG), a subregion of major interest in the context of antidepressant mechanisms. Transcriptome profiling on micro-dissected DG granule cells was performed to (i) reveal cell-type-specific changes in paroxetine-induced gene expression (paroxetine vs vehicle) and (ii) to identify molecular signatures of treatment response within a cohort of paroxetine-treated animals. We identified 112 differentially expressed genes associated with paroxetine treatment. The extreme group comparison (good vs poor responder) yielded 211 differentially expressed genes. General paroxetine effects could be distinguished from treatment response-associated molecular signatures, with a differential gene expression overlap of only 4.6% (15 genes). Biological pathway enrichment and cluster analyses identified candidate mechanisms associated with good treatment response, e.g., neuropeptide signaling, synaptic transmission, calcium signaling, and regulation of glucocorticoid secretion. Finally, we examined glucocorticoid receptor (GR)-dependent regulation of selected response-associated genes to analyze a hypothesized interplay between GR signaling and good antidepressant treatment response. Among the most promising candidates, we suggest potential targets such as the developmental gene *Otx2* or *Htr2c* for further investigations into antidepressant treatment response in the future.

## Introduction

Pharmacological treatment of major depressive disorder (MDD), the most prevalent mental illness worldwide with a still increasing burden of disease on Western societies^1^, faces several major drawbacks. First, the large heterogeneity of antidepressant treatment response leaves >30% of the patients without full remission of symptoms^2,3^. Second, we still lack clinically useful biomarkers that could either improve diagnostic accuracy, monitor disease state, or predict antidepressant response^4^. Third, a well-recognized shortcoming of currently available antidepressants is that significant difference from placebo can only be identified reliably some 6 weeks after initiating treatment, leading to “trial and error” approaches for each individual treatment decision^5^. Still, the neurobiological pathways that drive antidepressant response, in contrast to the average pharmacological drug effects, are far from being understood. Improving our knowledge about the neurobiological mechanisms shaping individual response to antidepressants could open up the opportunity to characterize putative novel targets mediating a more rapid onset of action.

Interestingly, heterogeneity in antidepressant response has not been systematically addressed in animal experimental approaches until very recently, although long being recognized as critical factor hampering antidepressant drug discovery and preclinical and clinical evaluation of potentially novel compounds. Previously, we have established and validated an approach to tackle this problem, which enables the selection of extreme phenotypes in an antidepressant-responsive mouse strain (DBA/2J; ref. ^6^). Specifically, when treating a large number of mice with the selective serotonin reuptake inhibitor (SSRI) paroxetine^7^, we were able to stratify them using the forced swim test (FST) and to select the extreme groups of good and poor antidepressant treatment responders, so as to model clinical heterogeneity in treatment outcomes as close as possible. We used this model to identify transcriptome signatures in peripheral murine blood, associated them with good treatment response, and validated those transcriptome signatures in cohorts of depressed patients. In addition, we showed that glucocorticoid receptor (GR) signaling played a pivotal role in mediating the differences between good and poor responders^7^.

The hippocampus is known as a key target for both antidepressant treatment^8^ and glucocorticoid effects^9^ on the brain. In addition, fine-tuning of the hypothalamic–pituitary–adrenal (HPA) axis—by modulating GR signaling—plays a crucial role in mediating recovery from depression^10–12^. Within the hippocampus, the dentate gyrus (DG) is a particularly interesting candidate region: antidepressants have been consistently shown to enhance adult neurogenesis in the DG granule cells, and antidepressant-like effects are strongly dependent on adult neurogenesis^13,14^. However, knowledge about the role of DG in the context of antidepressant treatment response as well as more detailed information on the genetic and epigenetic mechanisms mediating good and poor response to antidepressant treatment in this brain region are still lacking.

Here we analyzed (I) transcriptome changes in DG granule neurons induced by paroxetine treatment and (II) differences in DG transcriptome profiles between good and poor treatment responders, i.e., in a comparison of subgroups of extremes within a paroxetine-treated cohort of animals. This strategy allowed us to distinguish general paroxetine effects from treatment response-associated molecular signatures. To bridge the gap between HPA axis alterations, GR signaling, and mechanisms of antidepressant action, we finally addressed the question of whether our identified candidate response-associated genes are regulated in a GR-dependent manner.

## Materials and methods

### Animals

Male, adult (7–11 weeks) DBA/2J mice were purchased from Charles River (France). After arrival at our animal facility (temperature = 22 ± 2 °C, relative humidity = 50 ± 5 %), mice were single-housed and habituated to the new environment for at least 1 week prior to experimentation. We applied a standard dark/light cycle (8 a.m.–8 p.m. light, 8 p.m.–8 a.m. dark). Mice had ad libitum access to water and food. All experiments were conducted in accordance with the European directive 2010/63/EU for animal experiments and approved by the local animal welfare authority (Landesuntersuchungsamt Rheinland-Pfalz, Koblenz, Germany).

### Paroxetine treatment

The DBA/2J mouse strain has previously been shown to respond to oral treatment with the commonly used SSRI paroxetine under basal stress-free conditions^6^, and this was the most important argument in favor of paroxetine (5 mg/kg twice daily). The selection of this strain, with its well-known high innate anxiety and responsiveness to antidepressants, enabled us to perform the pharmacological treatment under basal conditions, i.e., without the need to subject the animals to an additional stress procedure that might have influenced the transcriptome data. A combination of stress exposure and antidepressant treatment within our approach would not allow us to identify the individual contribution of these two factors to the phenotype. To mimic the clinical conditions as close as possible, animals received customized palatable pills (40 mg PQPills, provided by I. Sillaber), with a concentration of 5 mg/kg body weight paroxetine hydrochloride (Sigma Aldrich, Germany) or vehicle^7^. We randomized the mice to treatment or control groups. Three days before the start of treatment, all mice were habituated to voluntary intake of vehicle pills given twice daily. Subsequently, mice received paroxetine or vehicle pills twice daily (8 a.m., 7 p.m.). Pill intake was monitored twice daily by screening the cages for remnants of pills. Mice that did not eat two or more pills over the treatment schedule were excluded from analyses.

### Paroxetine treatment effects on behavior and DG transcriptome profile

**Fig. 1:**
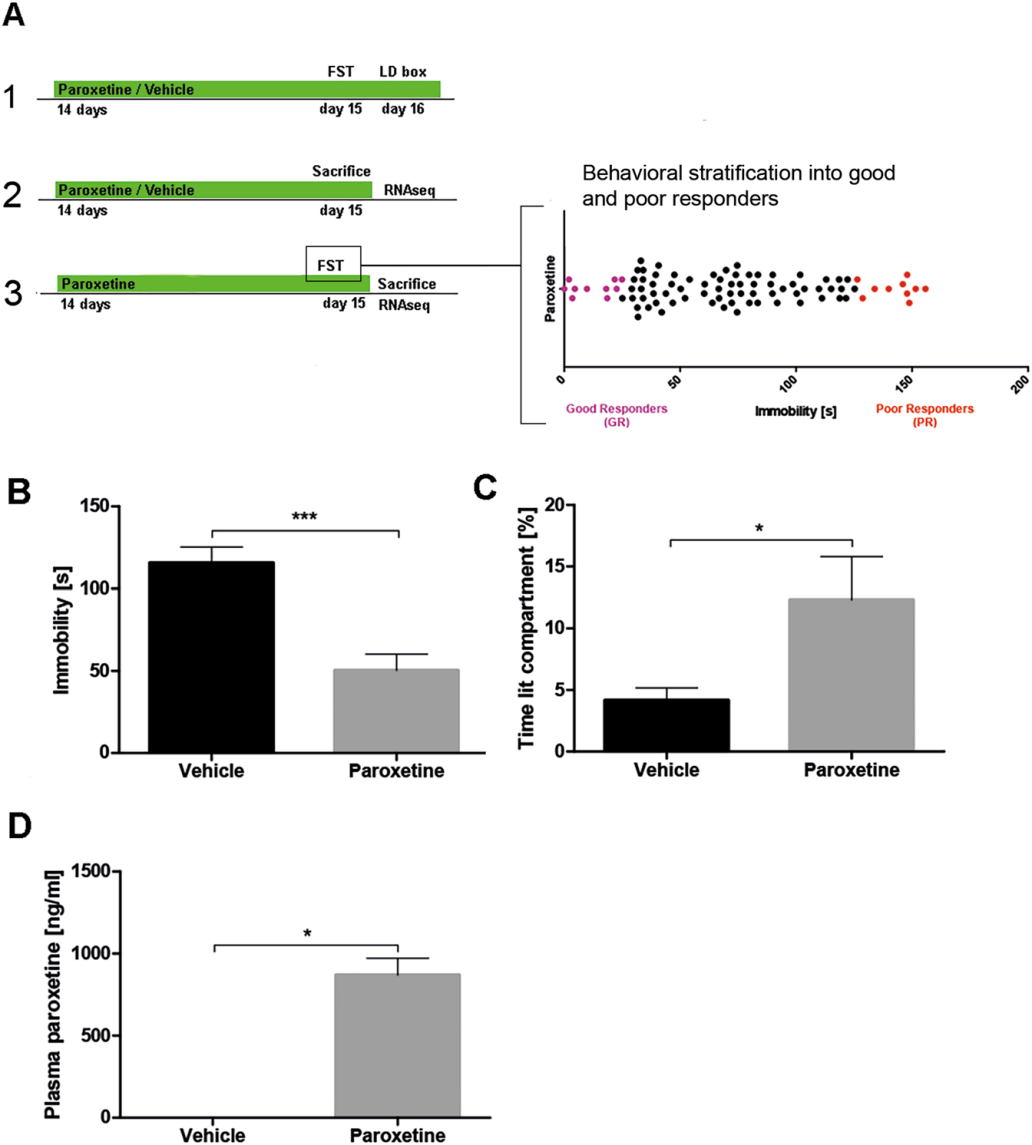
Experimental approach and effects of paroxetine treatment on depression-like behavior. **A** Schematic overview of the experimental schedules to assess the impact of paroxetine treatment on behavior (panel 1, *n* = 20) and dentate gyrus transcriptome profiles in comparison to vehicle-treated animals (panel 2, *n* = 13). To identify the molecular signatures underlying response, we stratified animals into good and poor treatment responder according to their behavioral outcome in the forced swim test (panel 3, *n* = 10 per subgroup good/poor responder). **B** Paroxetine treatment reduced immobility time (*T* test, *t* = 4.7, df = 18, *p* = 0.0002) in the FST in comparison to vehicle treatment. **C** Paroxetine treatment increased the time spent in the lit compartment of the LD box (*T* test, *t* = 2.2, df = 10, *p* = 0.0496). **D** Paroxetine plasma concentration was measured after treatment (Wilcoxon signed-rank test, *p* = 0.0313). FST forced swim test, LD light dark box, bars with mean and SEM. *p < 0.05; ***p < 0.001.

Mice were treated continuously with paroxetine or vehicle for 16 days (Fig. 1A, panel 1). On day 15 and 4 h after administration of paroxetine or vehicle in the morning, we performed the FST to assess depression-like behavior (paroxetine and control animals). On day 16 and 4 h after administration of paroxetine in the morning, the animals underwent the light dark (LD) test to assess anxiety behavior. To exclude uncontrollable effects of behavioral testing on DG transcriptome profiles, a separate batch of animals was used for RNA sequencing (RNAseq) analysis (Fig. 1A, panel 2). Mice were treated continuously with paroxetine for 15 days. Animals received the last administration of paroxetine in the morning of day 15. Four hours later, mice were sacrificed and single DG samples were dissected and stored at −80 °C for later RNA extraction and RNAseq (DG dissection and methods to ensure specificity of the dissection method, RNA extraction, and RNAseq are described in Supplemental Methods).

### Stratification of antidepressant treatment response and corresponding DG transcriptome profiles

A large batch of mice was treated continuously with paroxetine or vehicle pills for 15 days (Fig. 1A, panel 3). On day 15, 4 h after the last administration of paroxetine or vehicle in the morning, we conducted the FST. To exclude any putative influence of the FST on the transcriptional profile, all mice were sacrificed and hippocampal subregion DG was dissected and snap-frozen as described in the Supplemental Methods^15^. Mice were stratified into good and poor responders as previously described^7^. The 10 mice with lowest immobility time and the 10 with the highest immobility time were classified as good and poor responders, respectively. Ten vehicle-treated animals which also underwent FTS to ensure identical experimental conditions were taken as controls. Single DG samples were used for quantitative polymerase chain reaction (qPCR) and RNAseq experiments.

### Dexamethasone treatment and GR-dependent regulation of response-associated genes

In a new experiment with drug-naive animals, we conducted dexamethasone treatment as described by Arloth and colleagues^16^. From 8 to 11 a.m., mice were injected intraperitoneally with either 0.9% saline (Braun, Germany) or dexamethasone (Hexadreson®, MSD Animal Health, Germany) at 10 mg/kg body weight (Fig. 5A). Four hours after injection, mice were sacrificed and single DG samples were dissected and stored at −80 °C. Next, we extracted RNA and conducted qPCR.

### FST and LD box

We performed the FST as described in ref. ^7^ to assess depressive-like behavior. We introduced the mice into a 2-L glass beaker (diameter 13 cm, height 24 cm) filled with tap water (21 ± 1 °C) to a height of 15 cm. We videotaped the mice for 5 min and immobility time was scored by an experienced, treatment-blinded observer. We performed the LD box as described in ref. ^17^ to assess anxiety behavior. Mice were introduced into the lit compartment (600 lx) facing the wall and observed for 5 min. The time spent in the lit compartment was scored.

### Body weight, food intake, blood plasma collection, and corticosterone (CORT) plasma concentration

Body weight was measured at baseline and at the end of the experiment. Food intake was calculated by subtracting final food weight from baseline food weight. At baseline, we withdrew blood via tail cuts into EDTA-coated tubes. After sacrifice of the mice, trunk blood was collected in EDTA-coated tubes. We centrifuged all tubes for 10 min at 10,000 × *g* at 4 °C and kept the plasma stored at −80 °C until further steps were applied. Using blood plasma from RNAseq experiments, CORT plasma levels were measured with the Corticosterone ELISA Kit (Enzo Life Sciences, #Cat.No. ADI-900-0979) according to the manufacturer’s protocol in duplicates. Baseline CORT levels derived from blood plasma collections taken from 9 a.m. to 1 p.m., without any previous treatment. Final CORT levels were derived from blood plasma collections taken from trunk blood after 14 days of treatment (9 a.m.–-1 p.m.).

### Statistics

Sample sizes are indicated in the figure legends and were powered/calculated based on prior experiments. The values are displayed as mean ± SEM. Data were tested for normal distribution with the D’Agostino and Pearson omnibus normality test. Normally distributed data was analyzed using either the unpaired two-tailed *T* test, one- or two-way analysis of variance followed by Bonferroni correction. Mann–Whitney test was used for non-normally distributed data. Alpha was set at 5%, with *p* values < 0.05 considered statistically significant. Data were analyzed using the Prism 5 software (GraphPad, USA).

Additional information regarding DG dissection, RNA extraction, and molecular analyses can be found in the Supplemental Method section.

## Results

### Oral treatment with paroxetine reduces depression- and anxiety-like behavior

At the behavioral level, oral treatment with the SSRI paroxetine induced significant antidepressant-like and anxiolytic effects in DBA/2J mice (Fig. 1B, C). To validate successful paroxetine treatment, we measured paroxetine plasma concentrations in the animals (Fig. 1D). Paroxetine treatment also affected HPA axis function, eating behavior, and body weight: paroxetine-treated mice had lower plasma CORT levels (Fig. S1A), gained more body weight (Fig. S1B), and consumed more food (Fig. S1C) at the end of the study as compared to vehicle-treated mice.

### Molecular signature of paroxetine treatment in DG transcriptome profiles: stimulation of bone morphogenetic protein (BMP) and Hippo signaling pathways

**Fig. 2:**
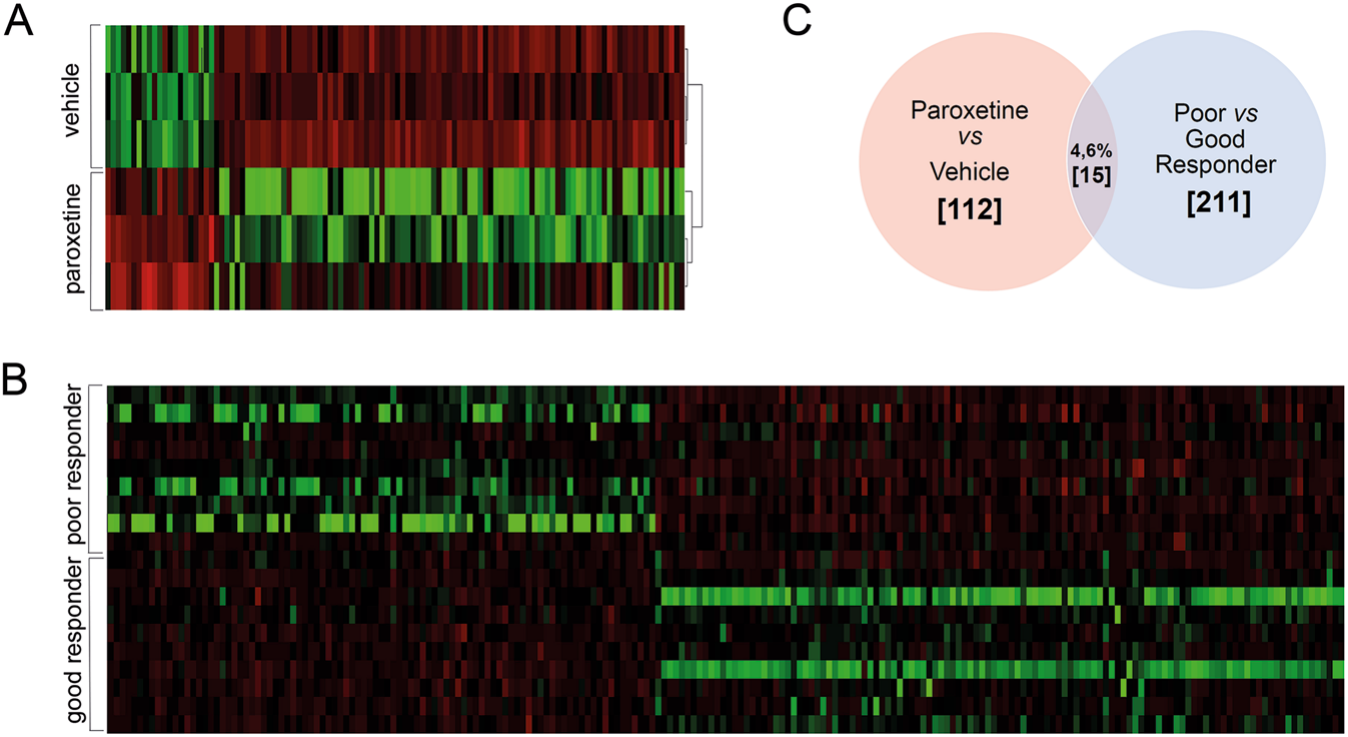
Effects of paroxetine treatment on dentate gyrus transcriptome profiles. **A** Heatmap illustrating the impact of paroxetine treatment in comparison to vehicle administration (control). In a 3 vs 3 comparison of murine dentate gyrus transcriptome profiles, we found 112 differentially expressed genes (DEseq: Normcount > 2.68 (avg), padj < 0.05, FC > 1.5 fold). **B** Dentate gyrus transcriptome profiles of good and poor responders: when comparing RNA extracted from dentate gryus of good vs poor responder mice, we identified 211 differentially expressed genes (DEseq: Normcount > 2.68 (avg), padj < 0.05, FC > 1.5 fold). **C** Overlap between treatment- and response-associated differentially expressed genes: we compared treatment- and response-associated differentially expressed genes and found only 15 (4.6%) of them to be differentially expressed in both comparisons. [*X*] indicates the number of differentially expressed genes in the respective comparisons in the Venn diagram.

First, we confirmed the specificity of DG dissection by qPCR of the DG-specific gene *TDO2* and the Cornu ammonis-specific gene *Lphn2* (Fig. S2). Using RNAseq analysis of DG tissue comparing paroxetine- and vehicle-treated animals, we revealed 91 upregulated differentially expressed genes (DEGs) and 21 downregulated DEGs (Fig. 2A). Protein–protein interaction (PPI) analysis showed three clusters of genes within the upregulated DEGs in the paroxetine–vehicle comparison (Fig. S3), covering the *Bmp*s, neurotransmitters, and collagens. In contrast, no clusters were identified in the downregulated DEGs. Biological enrichment analysis revealed 31 pathways associated with paroxetine treatment (Table S1), such as *BMP signaling pathway* and *Hippo signaling pathway*.

### Decoding the molecular pathways mediating response to antidepressant treatment: DG transcriptome profiles of good and poor responders

**Fig. 3:**
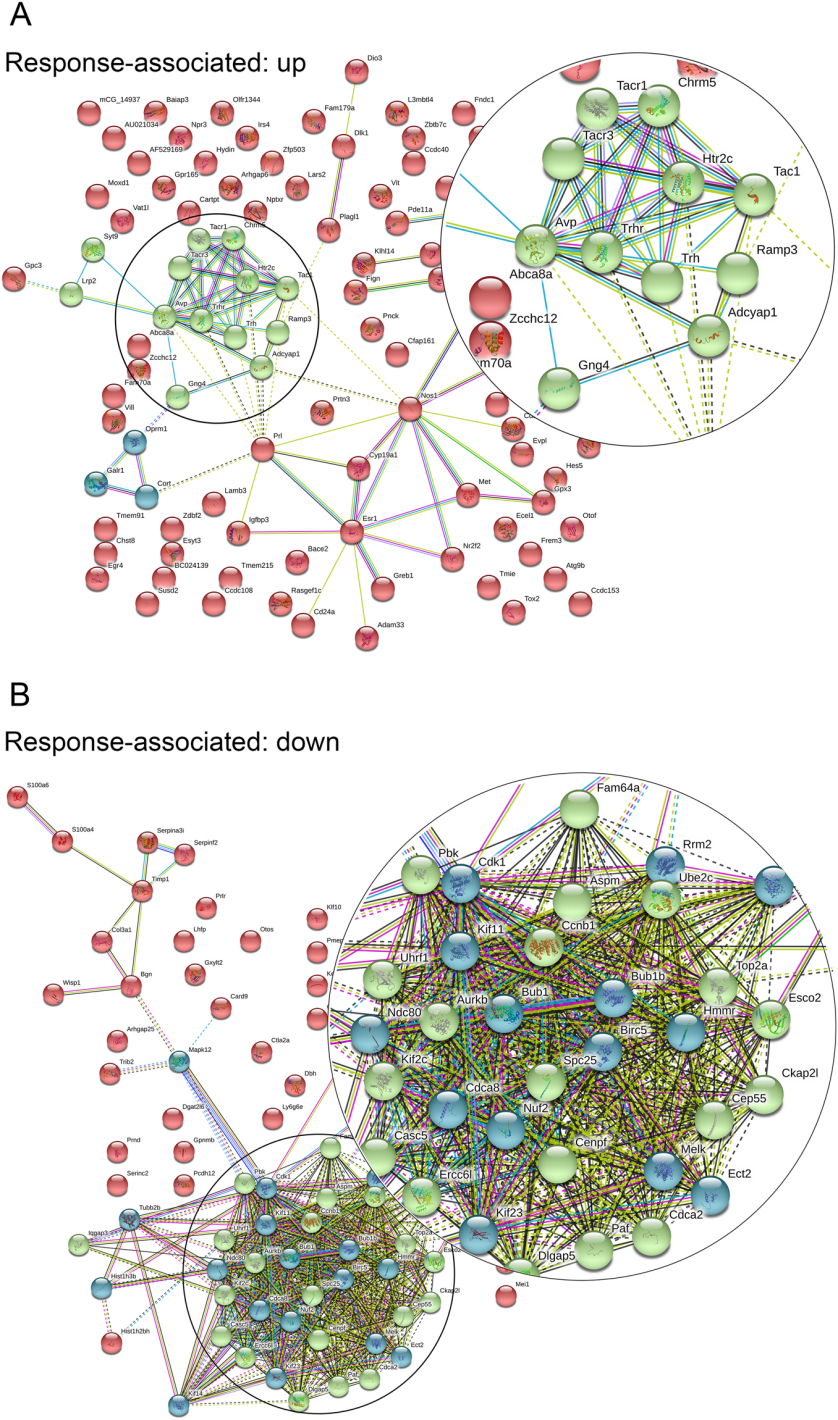
Pathway analyses of response-associated transcripts. **A** Upregulated response clusters: in a good vs poor responder comparison of dentate gyrus transcriptomes, we plotted all 117 upregulated genes and performed a kmeans clustering cluster analysis. We could detect two response-associated clusters (blue and green), and we showed their respective protein–protein interaction (PPI) profile (average local clustering coefficient: 0.304, PPI enrichment *p* value: <1.0e−16, *n* = 19). **B** Downregulated response clusters**:** in a good vs poor responder comparison of dentate gyrus samples, we plotted all 94 downregulated genes and performed a kmeans clustering cluster analysis. We could detect two response-associated clusters (blue and green) and we revealed their respective protein–protein interaction (PPI) profile (average local clustering coefficient: 0.528, PPI enrichment *p* value: <1.0e−16, *n* = 19). Insets show higher magnification of the most interesting clusters for improved visualization.

**Fig. 4:**
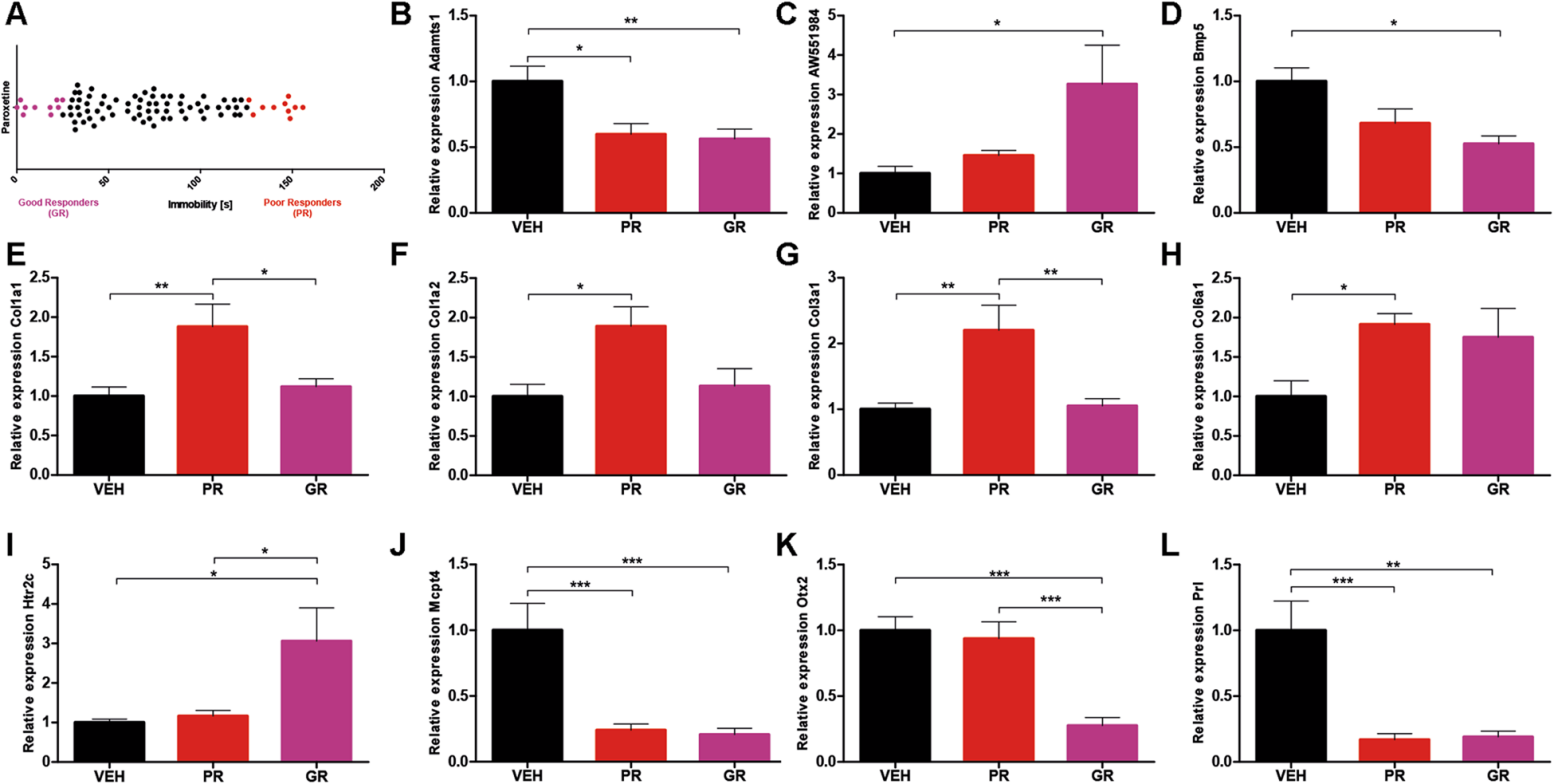
Validation of genes differentially expressed between of good and poor responders. **A** Paroxetine-treated animals and stratification in good and poor responder mice using the forced swim test (*n* = 90). **B**–**L** qPCR validation of 11 selected genes of interest (one-way ANOVA, Bonferroni’s Multiple Comparison Test). **B** *Adamts1*: *F* = 6.883, *p* = 0.0038. **C** *AW551984*: *F* = 3.917, *p* = 0.326. **D** *Bmp5*: *F* = 6.272, *p* = 0.0062. **E** *Col1a1*: *F* = 6.532, *p* = 0.0049. **F** *Col1a2*: *F* = 5.174, *p* = 0.0125. **G** *Col3a1*: *F* = 8.448, *p* = 0.0014. **H** *Col6a1*: *F* = 3.718, *p* = 0.0375. **I** *Htr2c*: *F* = 5.309, *p* = 0.0123. **J** *Mcpt4*: *F* = 12.39, *p* = 0.0002. **K** *Otx2*: *F* = 16.52, *p* < 0.0001. **L** *Prl*: *F* = 11.93, *p* = 0.0002. VEH vehicle, GR good responder, PR poor responder, bars with mean and SEM. *p < 0.05; **p < 0.01; ***p < 0.001.

Following 14 days of paroxetine treatment of DBA/2J mice, a considerable heterogeneity in the behavioral response to the treatment can be observed (Fig. 4A). In a good vs poor responder comparison, we identified a total of 211 genes to be differentially regulated, with 117 upregulated and 94 down-\regulated genes (Fig. 2B). PPI analysis revealed two clusters of genes within the upregulated DEGs (Fig. 3A) and two clusters of genes within the downregulated DEGs (Fig. 3B). Biological enrichment analysis revealed 46 pathways associated with response status (Table S2), such as *neuropeptide signaling pathway*, *synaptic transmission*, *calcium signaling*, and *positive regulation of glucocorticoid secretion*.

In an attempt to understand the differences between the transcriptome profiles of antidepressant treatment, i.e., average paroxetine effects, and response-associated profiles, we compared the 112 and 211 DEGs of the two RNAseq analyses. We could show that only 15 (4.6%) genes are differentially expressed in both experiments (Fig. 2C and Table 1).

**Table 1:** Overlap between treatment- and response-associated differentially expressed genes.

Gene name		Paroxetine vs vehicle	Good vs poor responder
Adenylate cyclase activating polypeptide 1	Adcyap1	↑	↑
AW551984	AW551984	↑	↑
Beta-site APP-cleaving enzyme 2	Bace2	↑	↑
Basonuclin 2	Bnc2	↑^a^	↓^a^
Cd24a	Cd24a	↑	↑
Muscarinic acetylcholine receptor M5	Chrm5	↑	↑
Dlk1	Dlk1	↑	↑
G protein-coupled receptor 101	Gpr101	↑	↑
5-hydroxytryptamine receptor 2C	Htr2c	↑	↑
Insulin-like growth factor binding protein 3	Igfbp3	↑	↑
Prolactin	Prl	↓^a^	↑^a^
Prolactin receptor	Prlr	↑^a^	↓^a^
Neuromedin-K receptor	Tacr3	↑	↑
Thyrotropin-releasing hormone receptor	Trhr	↑	↑
Vestigial like family member 3	Vgll3	↑	↑

We detected 15 treatment- and response-associated genes that were differentially expressed in both experiments. For 12 of them, gene expression was altered in the same direction ↑ upregulated gene, ↓ downregulated gene.

^a^Three of them (Bnc2, Prl, Prlr) showed the opposite regulation of gene expression.

Given the importance of HPA system modulation in depression and antidepressant treatment, we assessed HPA axis activity in vehicle-treated, good, and poor responder mice by plasma CORT measurement. At baseline, plasma CORT levels did not differ between vehicle-treated mice, good- and poor-responding animals. Both paroxetine-treated groups—good and poor responder mice—had significant lower plasma CORT levels than vehicle-treated mice 14 days post-treatment (Fig. S4). Baseline plasma concentrations of CORT did not predict later response status (data not shown).

Based on PPI, cluster, biological enrichment, and overlap analyses of DEGs from both experiments, we selected the 20 most interesting genes for validation by qPCR. We were able to detect statistically significant expression differences in 11 candidate genes (Fig. 4B–L). Within these genes, qPCR revealed a strong effect of paroxetine treatment, but at the same time no detectable difference was observed between good and poor response in 4 genes (*Adamts1*, *Bmp5*, *Mcpt4*, *Prl*; Figs. 4B and 2D, J, L). A significant difference between good responder and both vehicle and poor responder was found for *Htr2c* (Fig. 4I) and orthodenticle homeobox 2 (*Otx2*; Fig. 4K) qPCR. More precisely, *Htr2c* was upregulated in good responder mice, whereas *Otx2* was downregulated in good responder mice.

### Antidepressant treatment response-associated candidate genes are regulated by GR signaling

Based on the importance of GR signaling in modulating antidepressant treatment response, we investigated whether those 20 candidate genes identified in the transcriptome-wide approach are regulated in a GR-dependent manner. Specifically, there were two reasons to test the GR responsiveness of this subset of response-associated candidate genes: First, there is convincing evidence in the literature that response to antidepressants is modulated via the GR. Second, biological enrichment analysis for the genes differentially regulated between good and poor treatment response revealed, among others, Gene Ontology terms like *Response to steroid hormone*, *Positive regulation of glucocorticoid secretion*, and *Response to hormone* (see Supplemental Table 2) to be significantly enriched in the cluster of response-associated genes.

At the functional level, a more comprehensive way to test whether our antidepressant-response associated candidates are in fact responsive to GR activation would have been to integrate our RNAseq data with available datasets on GR-responsive genes, as we did in previous studies^7^. However, such a dataset was not available for the specific conditions that we used (e.g., DBA2J mouse strain, homogeneous cell population of DG granule neurons). Therefore, we tested the GR regulation in a targeted approach on a number of candidates that were pre-selected, as those candidates 20 were not “classic” GR-sensitive genes like, e.g., FKBP5, and conclusive information on their GR responsiveness was not available.

**Fig. 5:**
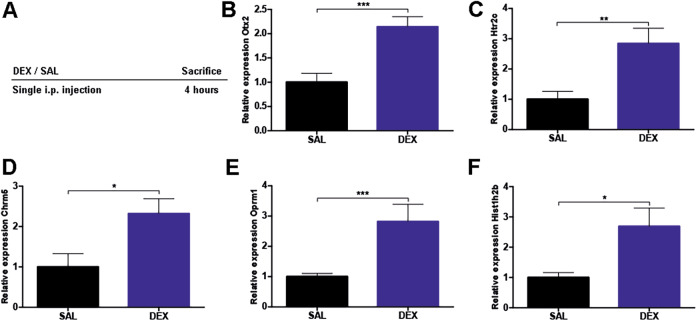
Response-associated genes are regulated by glucocorticoid receptor activation. **A** Schematic overview (*n* = 22). **B**–**F** qPCR of murine dentate gyrus samples revealed statistically significant upregulation of 5 genes (*T* test or Mann–Whitney test). **B** *Otx2*: *t* = 4.1, df = 18, *p* = 0.0006. **C** *Htr2c*: *t* = 3.3, df = 20, *p* = 0.0040. **D** *Chrm5*: *t* = 2.7, df = 14, *p* = 0.0182. **E** *Oprm1*: *U* = 3.0, *p* = 0.0003. **F** *Hist1h2b*: *t* = 2.8, df = 15, *p* = 0.0125. DEX dexamethasone, SAL saline, h hours, S sacrifice, bars with mean and SEM. *p < 0.05; **p < 0.01; ***p < 0.001.

To this end, gene expression in DG samples was analyzed by qPCR 4 h after injection of dexamethasone or saline. Five (Otx2, Htr2c, Chrm5, Oprm1, Hist1h2b) out of 20 response-associated genes were identified to be regulated by dexamethasone-induced GR activation (Fig. 5B–F).

## Discussion

Considering the urgent need to tailor individualized treatment strategies to fight depression more efficiently, a more detailed insight into the molecular mechanisms modulating antidepressant treatment response is fundamental. Here complementing human studies by strictly standardized animal experimental approaches can help to minimize biases and allows unrestricted access to the organ of interest, i.e., the brain.

Our approach to decode the molecular mechanisms underlying response to the SSRI paroxetine addresses the need to focus on individual outcomes rather than on average treatment effects. We hypothesized that focusing on the behavioral phenotype of individual treatment response might enable the identification of true response-associated molecular candidates, thus paving the way for the development of conceptually novel strategies to treat depression more efficiently in the future.

### The hippocampal DG: target of antidepressants and stress hormones

A large body of evidence suggests the hippocampal DG as a key target region of both antidepressant effects and stress-related processes. Since first reports by the Duman group in the year 2000^18^, it has been consistently shown that chronic treatment with antidepressants in general, and SSRIs in particular, stimulates multiple stages of adult neurogenesis in the DG (for a recent review, see ref. ^19^).

The first transcriptome profiling studies using early microarray techniques to identify antidepressant-induced RNA signatures were based on tissue homogenates of whole hippocampi^6,20^. Similarly, the majority of follow-up studies aiming to dissect the molecular pathways of antidepressant-like mechanisms did not focus on neuroanatomical subregions, cell populations, or circuits of the hippocampal formation. However, there is evidence showing the specific involvement of different hippocampal subregions and neuronal subpopulations in diverse and distinct functions of complex behavior^21^. In addition, a study by Anacker and colleagues, using an in vitro approach, could show that the antidepressant sertraline increases human hippocampal neurogenesis via a GR-dependent mechanism^22^. The specific involvement of DG granule cells in mediating antidepressant drug effects strongly supports the need for region specificity in the search for the molecular mechanism underlying antidepressants’ action. To ensure region and cell population specificity of our DG microdissection procedure, we confirmed that the dissected tissue was DG by conducting qPCR of the DG-specific gene tryptophan 2,3-dioxygenase (*TDO2*) for validation^15^. It is interesting to note that a recent analysis performed separately on dorsal and ventral sections from the DG revealed virtually identical and stable findings in antidepressant-induced gene expression states across the DG^23^. However, the overlap of the DEGs between this study and our experiment is small: only 19.8% of genes were also found to be differentially expressed by fluoxetine treatment in their study. This might be explained by the different antidepressant used or by differences in the experimental set-up: Samuels and colleagues performed several behavioral tests in the same animals that were used for RNA extraction, which might have affected gene expression^23^. Most importantly, however, is the fact that this study, but also other previous studies^6,20,23^, described transcriptional changes related to average treatment effects in the group but did not focus on the molecular pathways associated with the behavioral *response* (i.e., corresponding to individual good or poor response). We therefore conclude that the limited insight into the molecular mechanisms underlying individual response and improvement is one of the critical gaps of knowledge currently hampering the development of improved treatment strategies in depression.

### Focus on treatment response, not on mere paroxetine effects, reveals novel candidate transcriptional signatures

In line with our hypothesis, our data clearly show that the molecular signatures associated with treatment response in DG neurons are surprisingly distinct from those reflecting average drug treatment effects, with only 15 genes (4.6% of all DEGs) overlapping between the two RNAseq datasets (Fig. 2C and Table 1). In total, paroxetine treatment revealed 112 DEGs, whereas stratification into good and poor responders showed 211 DEGs (Fig. 2). This finding highlights the importance to optimize animal experimental models to mirror the clinical conditions as closely as possible and might help to explain why so far the search for molecular signatures shaping response to antidepressants has been relatively unsuccessful^24^. In line with our findings, a clinical study using the Genome-based Therapeutic Drugs for Depression study sample investigated biomarkers that are assessed before starting treatment and can predict future response to antidepressants (predictors), as well as biomarkers that are targeted by antidepressants and change longitudinally during the treatment (targets)^25^. Using a candidate-driven approach, Cattaneo and colleagues tested the leukocyte mRNA expression levels of genes belonging to GR function (e.g., FKBP-4, FKBP-5, and GR), several inflammatory markers and neuroplasticity-associated candidates (brain-derived neurotrophic factor, p11, and VGF). While this approach is different to the one undertaken in the current set of experiments in several aspects, it is interesting to note that the authors also found clear evidence for a dissociation between “predictors” and “targets” of antidepressant response. Moreover, both studies—although targeting completely different cell populations (hippocampal DG granule neurons vs peripheral blood leukocytes)—underline the importance of GR regulation for modulating the response to antidepressant treatment.

While there is a considerable number of animal models available aiming at mimicking disease (i.e., depression-like conditions), only a few preclinical or truly translational investigations are dedicated to the issue of heterogeneity in response to antidepressant treatment. Based on a recently described approach^7^, we have proposed a framework for improving translational studies into antidepressant treatment response^26^: By focusing on extreme subgroups within a relatively large cohort of inbred mice, we can select animals that respond extraordinarily well to paroxetine and compare those to a subgroup of animals, which are not distinguishable from vehicle-treated animals after chronic treatment with the antidepressant. Those good and poor responder subgroups closely model the clinical situation, where heterogeneity in antidepressant treatment outcomes is considerably large. A comparable strategy to focus on subgroups of animals displaying extremes in their phenotype has been pursued during the past years in animal models of stress and resilience research^27,28^ and has successfully enabled the identification of molecular signatures and interesting candidates of resilience^29^.

Using acetyl-L-carnitine (LAC) as a rapid-acting antidepressant agent, Bigio et al. analyzed the transcriptome profiles of ventral DG in a rat acute-stress model, and they also stratified their animals into responders and non-responders to the antidepressant compound^30^. Genes associated with energy metabolism were differentially regulated between LAC responders and non-responders^30^. With respect to the biological pathways involved, *response to estrogen*, *response to lipid*, and *response to steroid hormones* were pathways associated with response to LAC^30^. Although we used a conceptually different approach, a different species, and different antidepressant compound, we also identified the pathways *response to estrogen*, *response to lipid*, and *response to steroid hormones* to be associated with good response to antidepressant treatment (Table S2).

However, our analyses identified neuropeptide signaling, synaptic transmission, calcium signaling, and regulation of glucocorticoid secretion (Table S2) as additional pathways to be significantly associated with antidepressant treatment response in the DG. The comparison of paroxetine treatment vs vehicle resulted in differentially regulated clusters of genes, namely, a neurotransmitter and neurotransmitter receptor cluster (Fig. S3 green), a BMP signaling cluster (Fig. S3 blue), and a collagen cluster (Fig. S3 yellow). The good vs poor responder comparison identified two DEG clusters (Fig. 3). Our data are in line with previous reports on transcriptome signatures of antidepressant treatment, where hippocampal BMP signaling was recently reported to play a role in antidepressant treatment^31^. Indeed, both the BMP signaling pathway (Table S1) and *Bmp7* (Fig. 2) were regulated in our paroxetine vs vehicle experiment, but we did neither confirm BMP signaling nor *Bmp7* to be differentially regulated when focusing on response in the phenotypic readout.

Molecular targets that we considered particularly interesting for further discussion and investigation are those for which (a) we detected a significant difference between good responder and both vehicle and poor responder groups, and (b) regulation of gene expression could be proven to be GR responsive. One of those targets of particular interest that we identified to be exclusively upregulated in good responder mice with no change of expression level in poor responders (Fig. 4I) is the serotonin receptor type 2C, *Htr2c* gene. Interestingly, cluster analysis revealed *Htr2c* as a central hub within one of the treatment-associated clusters of the upregulated genes (Fig. S3). The brain serotonin system is the primary target of SSRIs, such as paroxetine, and there is solid evidence to support that *Htr2c* is critically involved in modulating synaptic plasticity, neuronal activity, and anxiety-related behavior^32–34^. After 2 weeks of fluoxetine treatment, *Htr2c* mRNA levels were upregulated in the hippocampus of rodents^35^. Clinical studies revealed that human *Htr2c* polymorphisms increased susceptibility to MDD^36^ and found *Htr2c* polymorphism *rs6318* to be associated with response in men^37^. In addition, we provide first evidence that *Htr2c* is directly regulated via a GR-dependent mechanism, as treatment with the GR-agonist dexamethasone significantly increased *Htr2c* expression (Fig. 5C), thus highlighting a possible connection between GR signaling and antidepressant response. Indeed, *Htr2c* is necessary for the communication between serotonin and the HPA axis^32^, and genetic variants within *Htr2c* have been shown to modulate the magnitude of the stress response in humans^38,39^. This combined evidence suggests *Htr2c* as an interesting candidate for further experiments investigating neurobiological mechanisms underlying antidepressant treatment response in general and its molecular link to fine-tuning of the HPA system in particular.

### Response to antidepressants involves factors related to experience-dependent plasticity: the role of *Otx2*

The homeobox gene *Otx2* is classically known to play a critical role as transcription factor in neurodevelopment where it is involved in the regional patterning of the midbrain and forebrain^40^. Besides its prominent role in neurodevelopment, *Otx2* has been more and more recognized as a main mediator of neuronal and experience-related plasticity^41^ and has recently been found to modulate depression in humans^42^ and stress-related outcomes in animal models^43^. In this latter study, Pena et al. did not identify Otx2 directly to be regulated by their early-life stress paradigm, but Otx2 was identified as a putative upstream regulator via pathway analyses of regulated transcripts This means that lasting effects of early-life stress directly on Otx2 could not be detected. Yet, loss-of-function and gain-of-function studies provided evidence that induction of Otx2 can rescue negative outcomes of early-life stress. When comparing these findings to the present set of data, it is important to consider that Pena and colleagues were targeting effects of a stressful manipulation that occurred during a particular postnatal development, i.e., a window during which the endogenous expression levels and regulation of Otx2 are different from adulthood, and during which also the regulation of the stress hormone system is different from adult stages. In adult animals, the presence of *Otx2* suppresses plasticity, whereas its inactivation or absence allows plasticity to occur^44^. Under physiological conditions, *Otx2* might thus help to consolidate or maintain memories over time. However, experience-dependent plasticity might be a crucial mechanism to successfully cope with environmental stressors and challenges or to recover from depressive states. In accordance with this notion, we found *Otx2* expression in the DG to be exclusively downregulated in good responders, with no change of expression level in bad responders (Fig. 4K). In line with that, we observed a high number of upregulated genes encoding extracellular matrix proteins in good responders, possibly indicating a higher capability of cellular and structural remodeling (Table S2). In addition, *Otx2* expression was increased by dexamethasone treatment (Fig. 5B), indicating a possible relevance of GR responsiveness in the *Otx2*-associated response to antidepressant treatment. While epidemiological data already suggest that a history of early-life trauma predicts poorer response to antidepressant therapy^45^, further mechanistic studies are needed to dissect the putative role of stress experiences in exerting a lasting impact on *Otx2* regulation. Considering its prominent role in neurodevelopment, timing of stress and glucocorticoid exposure might be critical in shaping their impact on *Otx2* function.

## Conclusion

In summary, our data provide novel insights into the molecular pathways mediating response to treatment with the SSRI paroxetine, as we identified distinct transcriptome signatures associated with antidepressant response in DG granule neurons. The unexpected discrepancy between treatment- and response-associated transcriptome signatures underlines the importance of refining animal experimental approaches for neuropsychiatric drug discovery. The identification of novel plasticity candidate genes involved in modulation of treatment response supports the hypothesis that a successful antidepressant treatment needs to re-open a particular window enabling experience-dependent plasticity for recovery from depression. Specifically, candidates such as *Otx2* might provide the molecular basis to explain why there is solid evidence supporting the fact that, for the majority of patients with MDD, a combination of pharmacotherapy together with cognitive behavioral therapy is superior in antidepressant efficacy than pharmacotherapy or psychotherapy alone. Antidepressants need to be combined with psychological training to reorganize networks rendered more plastic by the drug treatment^46^.

## Supplementary information

Supplemental figures and tables

Supplemental methods
