# Supplementary material for: A distinct transcriptional signature of antidepressant response in hippocampal dentate gyrus granule cells
Source: Transl Psychiatry. 2021 Jan 5;11:4. doi: 10.1038/s41398-020-01136-2 (PMC7791134; doi:10.1038/s41398-020-01136-2)
Supplement: Supplementary file 1 — Supplemental figures and tables [file 41398_2020_1136_MOESM1_ESM.docx]

**Supplemental figures and tables**

Content 1

Figure S1 – Effects of paroxetine treatment on HPA axis activity and nutrition 2

Figure S2 - Validation of dentate gyrus (DG) dissection technique 3

Figure S3 - Paroxetine treatment clusters 4

Table S1 - Biological enrichment analysis of paroxetine treatment in murine DG samples 5

Table S2 - Biological enrichment analysis of good and poor responders in murine DG samples 6

Figure S4 - HPA axis activity across good and poor responders 8

**A B C**


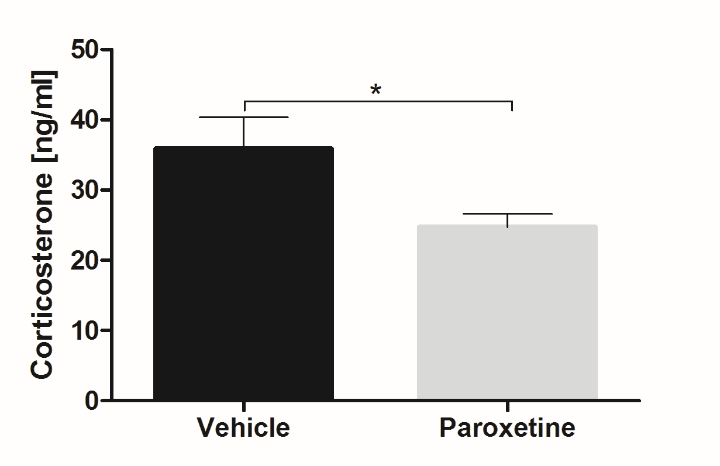

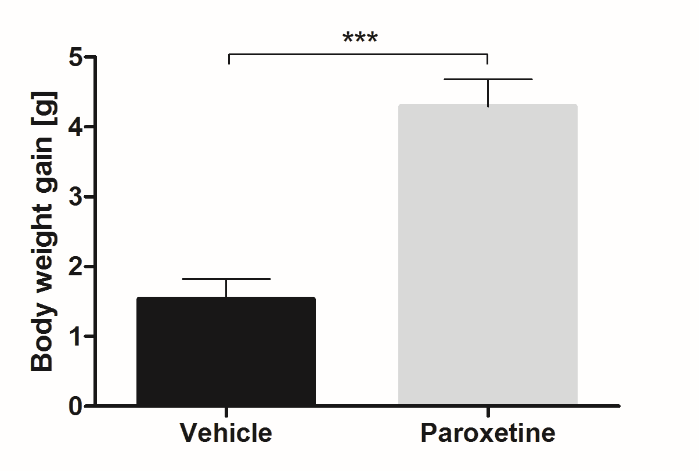

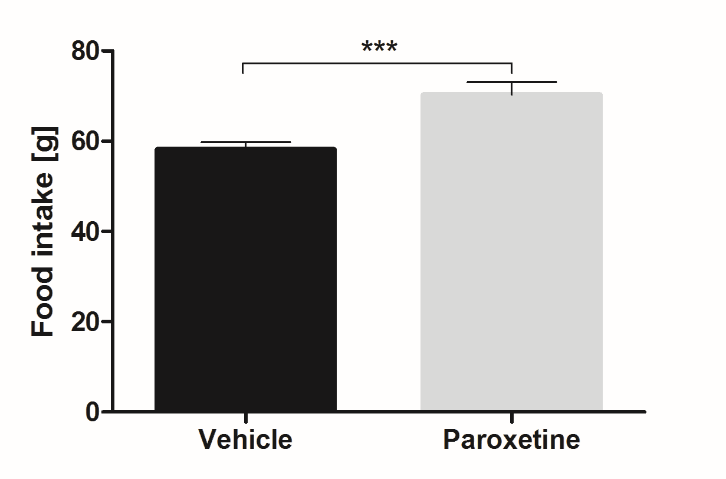


**Figure S1 - Effects of paroxetine treatment on HPA axis activity and food intake.** For 16 days, mice were treated with either vehicle or paroxetine. At the end of the experiment, we measured plasma corticosterone concentration (A), body weight (B), and food intake (C). Two weeks of paroxetine treatment produced a statistically significant decrease of corticosterone concentration (T test, t=2.745, df=24, p=0.0113, n=36), an increase in body weight gain (Mann Whitney test, U=18.00, p<0.0001, n=32), and an increase in food intake (T test, t=3.865, df=30, p=0.0006, n=32). Bars with mean and SEM.


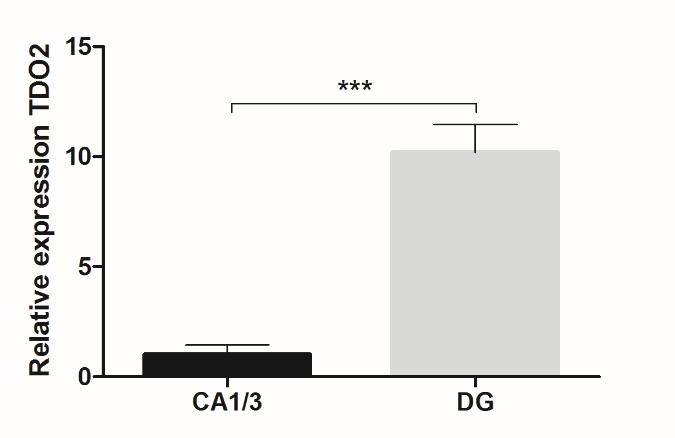

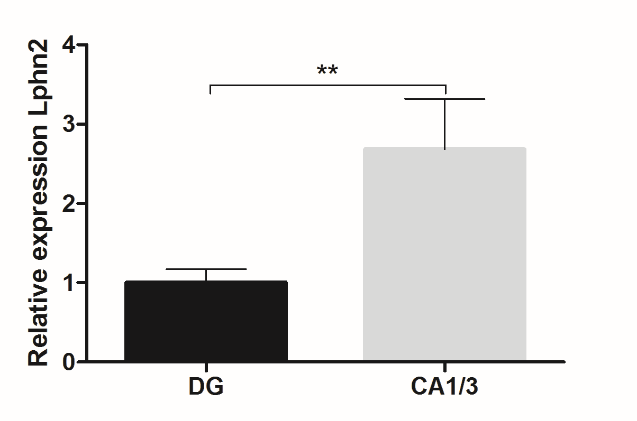


**Figure S2 – Validation of dentate gyrus (DG) dissection technique.** We validated the specificity of DG dissection by qPCR of DG-specific gene *TDO2* and CA-specific gene *Lphn2*. We found a statistically significant higher *TDO2* expression (Mann Whitney test, U=2.000, p<0.0001) in DG samples compared to the rest of the hippocampus (CA1/3). We found a significant higher *Lphn2* expression (Mann Whitney test, U=17.00, p=0.0048) in the rest of the hippomcapus (CA1/3) compared to DG samples. Data are normalized to the control (=1.0), bars with mean and SEM, n=10, DG dentate gyrus, CA cornu ammonis.


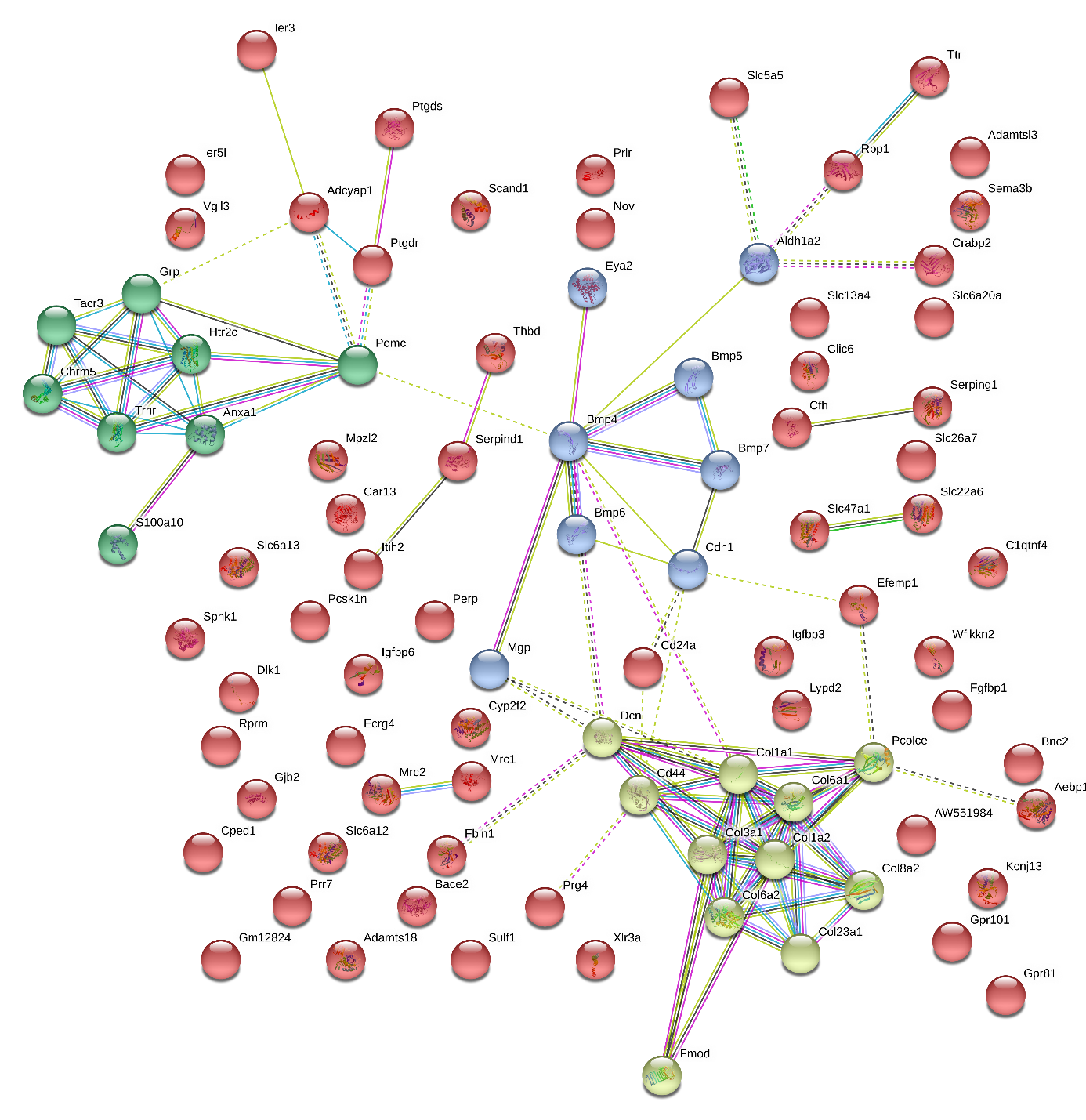


**Figure S3 - Paroxetine treatment clusters.** In a 3 vs 3 comparison of dentate gyrus samples, we plotted all 91 up-regulated genes and performed a kmeans clustering cluster analysis. We could detect three paroxetine treatment-associated clusters (green, blue, and yellow) and we showed their respective protein-protein interaction (PPI) profile (average local clustering coefficient: 0.407, PPI enrichment p-value < 1.0e-16).

| **Biological enrichment analysis – Paroxetine vs vehicle** | | |
| --- | --- | --- |
| **Gene Ontology Biological Process - Term** | **Genes** | **p value** |
| Positive regulation of transcription from RNA polymerase II promoter | 12 | 4.72E-05 |
| Cell-cell signaling | 5 | 1.64E-04 |
| Positive regulation of transcription, DNA-templated | 8 | 7.88E-04 |
| Negative regulation of chondrocyte differentiation | 3 | 0.00102236 |
| SMAD protein signal transduction | 4 | 0.00111075 |
| Cell adhesion | 7 | 0.00168326 |
| Growth | 3 | 0.00408387 |
| Response to estradiol | 4 | 0.00471732 |
| Positive regulation of pathway-restricted SMAD protein phosphorylation | 3 | 0.00716598 |
| Cellular response to hormone stimulus | 3 | 0.00805914 |
| Embryonic skeletal system morphogenesis | 3 | 0.00932371 |
| Positive regulation of cell proliferation | 6 | 0.01404564 |
| Cell fate commitment | 3 | 0.01559748 |
| Positive regulation of epithelial cell proliferation | 3 | 0.01727562 |
| Negative regulation of cell death | 3 | 0.01947626 |
| Cartilage development | 3 | 0.01992986 |
| Protein processing | 3 | 0.02178829 |
| BMP signaling pathway | 3 | 0.02226378 |
| Angiogenesis | 4 | 0.02587727 |
| Negative regulation of canonical Wnt signaling pathway | 3 | 0.03098521 |
| Skeletal system development | 3 | 0.03264431 |
| Positive regulation of angiogenesis | 3 | 0.04144293 |
| Lung development | 3 | 0.04455353 |
| Negative regulation of cell growth | 3 | 0.04455353 |
| **Kyoto Encyclopedia of Genes and Genomes - Pathway** | **Genes** | **p value** |
| Hippo signaling pathway | 5 | 0.00110169 |
| TGF-beta signaling pathway | 4 | 0.00220624 |
| Basal cell carcinoma | 3 | 0.01244139 |
| Prolactin signaling pathway | 3 | 0.02128297 |
| Herpes simplex infection | 4 | 0.02572101 |
| Pathways in cancer | 5 | 0.03233499 |
| Melanogenesis | 3 | 0.03741335 |

**Table S1 – Biological enrichment analysis of paroxetine treatment in murine DG samples.** We analyzed differentially expressed genes (paroxetine vs vehicle, n=6) and highlighted all statistically significant enrichments.

| **Biological enrichment analysis - good vs poor responder** | | |
| --- | --- | --- |
| **Gene Ontology Biological Process - Term** | **Genes** | **p value** |
| Neuropeptide signaling pathway | 9 | 8.9E-06 |
| Cytosolic calcium ion homeostasis | 9 | 0.00052 |
| Positive regulation of cytosolic calcium ion concentration | 8 | 0.00092 |
| Synaptic transmission | 11 | 0.00092 |
| G-protein coupled receptor signaling pathway | 16 | 0.00113 |
| Cell-cell signaling | 13 | 0.00208 |
| Circulatory system process | 9 | 0.00382 |
| Cell communication | 36 | 0.00382 |
| Adenylate cyclase-modulating G-protein coupled receptor signaling pathway | 7 | 0.00382 |
| Positive regulation of signaling | 19 | 0.00502 |
| Regulation of blood pressure | 7 | 0.00523 |
| Phospholipase C-activating G-protein coupled receptor signaling pathway | 5 | 0.00583 |
| Single organism signaling | 34 | 0.00583 |
| Response to morphine | 4 | 0.00596 |
| Response to organic cyclic compound | 14 | 0.00605 |
| Signal transduction | 32 | 0.00909 |
| Response to alcohol | 9 | 0.00909 |
| Response to alkaloid | 6 | 0.00988 |
| Blood circulation | 8 | 0.0125 |
| Regulation of cyclic nucleotide biosynthetic process | 6 | 0.0125 |
| Response to estrogen | 7 | 0.0135 |
| Response to ammonium ion | 5 | 0.0135 |
| Regulation of purine nucleotide biosynthetic process | 6 | 0.0135 |
| Regulation of cyclase activity | 5 | 0.0169 |
| Positive regulation of blood pressure | 4 | 0.0169 |
| Regulation of lyase activity | 5 | 0.0184 |
| Cellular chemical homeostasis | 10 | 0.0184 |
| Regulation of system process | 9 | 0.0186 |
| Positive regulation of cell communication | 18 | 0.02 |
| Negative regulation of adenylate cyclase activity | 3 | 0.0232 |
| Regulation of hormone secretion | 7 | 0.0232 |
| Adenylate cyclase-inhibiting G-protein coupled receptor signaling pathway | 4 | 0.0254 |
| Regulation of renal sodium excretion | 3 | 0.0254 |
| Response to steroid hormone | 9 | 0.0254 |
| Response to hormone | 12 | 0.0285 |
| Single-organism behavior | 9 | 0.0302 |
| Positive regulation of hormone secretion | 5 | 0.0334 |
| Response to lipid | 12 | 0.0343 |
| Positive regulation of saliva secretion | 2 | 0.035 |
| Positive regulation of glucocorticoid secretion | 2 | 0.035 |
| Regulation of cAMP biosynthetic process | 5 | 0.0357 |
| Regulation of neurological system process | 4 | 0.0413 |
| Regulation of phosphate metabolic process | 17 | 0.0486 |
| Hormone metabolic process | 5 | 0.0486 |
| Regulation of blood circulation | 6 | 0.0486 |
| **Kyoto Encyclopedia of Genes and Genomes - Pathway** | **Genes** | **p value** |
| Neuroactive ligand-receptor interaction | 10 | 0.00034 |
| **Calcium signaling pathway** | **6** | **0.034** |

**Table S2 – Biological enrichment analysis of good and poor responders in murine DG samples.** We analyzed differentially expressed genes (good vs poor responder, n=19) and highlighted all statistically significant enrichments.


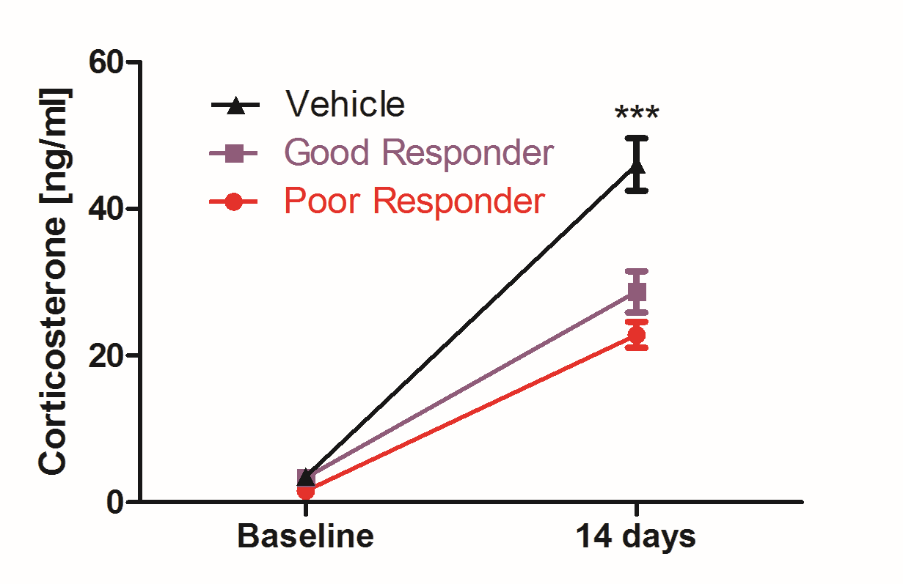


**Figure S4 - HPA axis activity across good and poor responders.** We measured plasma corticosterone levels at baseline and 14 days after paroxetine or vehicle treatment. Plasma corticosterone levels did not differ at baseline. Paroxetine treatment led to statistically significant lower corticosterone levels compared to vehicle-treated mice (2way ANOVA: time point, F=130.5, df=1, p<0.0001; treatment, F=8.208, df=2, p=0.0005; interaction, F=6.276, df=2, p=0.0028). There was no difference between good and poor responders (Bonferroni posttest: t=1.152, p>0.05). Data presented with mean and SEM, n=49.
