# Supplementary material for: A distinct transcriptional signature of antidepressant response in hippocampal dentate gyrus granule cells
Source: Transl Psychiatry. 2021 Jan 5;11:4. doi: 10.1038/s41398-020-01136-2 (PMC7791134; doi:10.1038/s41398-020-01136-2)
Supplement: Supplementary file 2 — Supplemental methods [file 41398_2020_1136_MOESM2_ESM.docx]

**Supplemental methods**

Content 1

Paroxetine plasma concentration 2

Microdissection of the dentate gyrus from native brain tissue 2

RNA extraction and quantitative real time RT-PCR (qPCR) 2

RNA sequencing (RNAseq) and data analysis 3

References for supplemental methods 4

*Paroxetine plasma concentration*

Plasma samples were submitted to a high-performance liquid chromatography system (Agilent 1200). Samples were loaded at 25°C and subjected to 5 minutes of extraction and 15 minutes of separation. We used the extraction column PerfectBond CN 20µm, 20x4mm (MZ-Analysentechnik, Germany) and the analytic column LiChrospher 60 RP-Select B, 5µm, 125x4mm (MZ-Analysentechnik, Germany) to measure paroxetine plasma concentration.

*Microdissection of the dentate gyrus from native brain tissue*

Mice were rapidly anesthetized with isoflurane and decapitated. On ice, we quickly removed the brains and micro-dissected both hemispheres separately to get single DG samples using a stereoscopic dissection microscope (Leica, Germany) as previously described [1]. Subsequently, we immediately froze single DG samples and stored them at -80°C. In preliminary experiments, we successfully validated the specificity of the dissection technique (Figure S2) as recommended by [1].

*RNA extraction and quantitative real time RT-PCR (qPCR)*

RNA was extracted from single DG samples by a combination of the RNeasy Micro Kit (Qiagen, Germany) in combination with TRIzol® (ThermoFisher, Germany). The method is described elsewhere [2], but in the present study we used single DG samples instead of brain punches, randomized for brain laterality. Quality control of RNA samples was performed using Qubit® (ThermoFisher, Germany) concentration measurement and Bioanalyzer profiling (Agilent Technologies, Germany). Concentrations were >100ng/µl, with RIN values >8.0. 500ng of RNA was used to synthesize cDNA according to the manufacturers’ protocol with the PrimeScript™ RT Master Mix (Takara Bio Inc., Japan). We performed qPCR using the SYBR® Green PCR Master Mix (ThermoFisher, Germany) and a StepOnePlus qPCR device (ThermoFisher, Germany). Primer pairs were customized using primer-BLAST (https://www.ncbi.nlm.nih.gov/tools/primer-blast/) and ordered from Sigma Aldrich (Germany). Primer specificity was tested with melting curves and agarose gels before use. PCR cycling conditions were: 15min at 95°C; [15s at 94°C, 30s at 55°C, 30s at 72°C] x 40 cycles. Two housekeeping genes (*HPRT1* and *GAPDH*) were used to normalize mRNA of genes of interest using the ∆∆ct method. Data was normalized and compared to control values set at 1.0.

*RNA sequencing (RNAseq) and data analysis*

Paroxetine treatment RNAseq: Next-generation sequencing (NGS) library prep was performed with Illumina's TruSeq stranded mRNA LT Sample Prep Kit following Illumina’s standard protocol (Part # 15031047 Rev, E). Libraries were prepared with a starting amount of 500 ng and amplified in 11 PCR cycles. Libraries were profiled in a DNA 1000 Chip on a 2100 Bioanalyzer (Agilent technologies) and quantified using the Qubit dsDNA HS Assay Kit, in a Qubit 2,0 Fluorometer (Life technologies). All libraries were pooled in equimolar ratio and sequenced on 3 rapid HiSeq 2500 lanes, SR for 1x 68 cycles plus 7 cycles for the index read, obtaining on average 35 million reads per library.

Good and poor responder RNAseq: NGS library prep was performed with Illumina's TruSeq stranded Total RNA LT Sample Prep Kit following Illumina’s standard protocol (Part # 15031048 Rev. E). Libraries were prepared with a starting amount of 114ng and amplified in 15 PCR cycles. Libraries were profiled in a DNA 1000 chip on a 2100 Bioanalyzer (Agilent technologies) and quantified using the Qubit dsDNA HS Assay Kit, in a Qubit 2.0 Fluorometer (Life technologies). All libraries were pooled together in equimolar ratio and sequenced on 8 NextSeq 500 Highoutput FC, SR for 1x 75 cycles plus 16 cycles for the index reads (8 + 8), obtaining on average 40 million reads per library.

RNAseq raw fastq files were aligned to the mouse reference genome (mm9) using TopHat [3] and the transcript count was calculated using HTSeq [4]. A principle component analysis was applied to exclude gene expression outliers. Based on transcript count, differentially expressed genes (DEGs) across the conditions were identified using DESeq package [5]. The expression cutoff of the genes were defined based on the density plot of log_2_ normalized read count. The criteria for the significant differential expression was defined as the padj < 0.05 and the absolute log_2_-fold change more than 0.58, and the average normalized read count larger than the expression cutoff. Expression levels of DEGs were plotted and graphically displayed as a heatmap using the online tool Heatmapper [6]. Protein-protein interactions and biological enrichment of the DEGs were analyzed using the bioinformatics tool STRING [7]. Datasets were uploaded and can be accessed using doi:10.5061/dryad.5mkkwh74d.

*References:*

1. Hagihara H, Toyama K, Yamasaki N, Miyakawa T. Dissection of hippocampal dentate gyrus from adult mouse. Journal of visualized experiments : *JoVE.* **33**. doi:10.3791/1543 (2009).

2. Jene T, Gassen NC, Opitz V, Endres K, Müller MB, van der Kooij MA. Temporal profiling of an acute stress-induced behavioral phenotype in mice and role of hippocampal DRR1. *Psychoneuroendocrinology*. **91**:149-58. doi:10.1016/j.psyneuen.2018.03.004 (2018).

3. Trapnell C, Pachter L, Salzberg SL. TopHat: discovering splice junctions with RNA-Seq. *Bioinformatics*. **25**(9):1105-11. doi:10.1093/bioinformatics/btp120 (2009).

4. Anders S, Pyl PT, Huber W. HTSeq--a Python framework to work with high-throughput sequencing data. *Bioinformatics*. 31(2):166-9. doi:10.1093/bioinformatics/btu638 (2015).

5. Anders S, Huber W. Differential expression analysis for sequence count data. *Genome Biol.* **11**(10):R106. doi:10.1186/gb-2010-11-10-r106 (2010).

6. Babicki S, Arndt D, Marcu A, Liang Y, Grant JR, Maciejewski A, et al. Heatmapper: web-enabled heat mapping for all. *Nucl. Acids Res*. **44**(W1):W147-53. doi:10.1093/nar/gkw419 (2016).

7. Szklarczyk D, Morris JH, Cook H, Kuhn M, Wyder S, Simonovic M, et al. The STRING database in 2017: quality-controlled protein-protein association networks, made broadly accessible. *Nucl. Acids. Res*. **45**(D1):D362-D8. doi:10.1093/nar/gkw937 (2017).
